# A Qualitative Study Comparing the Assay Performance Characteristics Between the 2007 and the 2013 American Society for Clinical Oncology and College of American Pathologists HER2 Scoring Methods in Mucinous Epithelial Ovarian Cancer

**DOI:** 10.1097/MD.0000000000000171

**Published:** 2014-12-12

**Authors:** Chi-Kuan Chen, Ming-Yung Lee, Wea-Lung Lin, Yu-Ting Wang, Chih-Ping Han, Cheng-Ping Yu, Wan-Ru Chao

**Affiliations:** From the Graduate Institute of Life Sciences, National Defense Medical Center (C-KC); Department of Pathology, Laboratory Medicine (C-KC); Department of Medicine, Mackay Medical College, Taipei (C-KC); Department of Statistics and Informatics Science, Providence University (M-YL); Department of Pathology (W-LL, Y-TW, C-PH, W-RC); Department of Obstetrics and Gynecology, School of Medicine, Chung-Shan Medical University and Chung-Shan Medical University Hospital, Taichung (C-PH); Graduate Institute of Life Sciences (C-PY); Department of Pathology, Tri-Service General Hospital, National Defense Medical Center, Taipei (C-PY); and Institute of Medicine, Chung-Shan Medical University, Taichung, Taiwan (W-RC).

## Abstract

The remarkable success of trastuzumab and other newly developed anti-HER2 (human epidermal growth factor receptor 2) therapies in breast, gastric, or gastroesophageal junction cancer patients has supported us to investigate the HER2 status and its possible therapeutic implication in mucinous epithelial ovarian cancer (EOC). However, there is currently no standardization of HER2 scoring criteria in mucinous EOC. In this study, we aimed to compare both the assay performance characteristics of the 2007 and the 2013 American Society for Clinical Oncology and College of American Pathologists scoring methods. Forty-nine tissue microarray samples of mucinous EOC from Asian women were analyzed by immunohistochemistry (IHC) and fluorescence in situ hybridization (FISH) tests using the 2007 and the 2013 criteria, respectively. The overall concordance between IHC and FISH by the 2007 criteria was 97.92 % (kappa = 0.921), and that by the 2013 criteria was 100% (kappa = 1.000). The percentage of *Her2* FISH-amplified cases showed an increasing trend significantly through their corresponding HER2 IHC ordinals by the 2007 and the 2013 criteria, respectively (*P* < 0.001, *P* < 0.001). After excluding equivocal cases, the specificity (100%) and positive predictive value (100%) were unchanged under either the 2007 or the 2013 criteria. The sensitivity (100%), negative predictive value (NPV) (100%), and accuracy (100%) of HER2 IHC were higher under the 2013 criteria than those (sensitivity 87.5%, NPV 97.6%, and accuracy 97.9%) under the 2007 criteria. Of the total 49 cases, the number (n = 4) of HER2 IHC equivocal results under the 2013 criteria was 4-fold higher than that (n = 1) under the 2007 criteria (8.16% vs 2.04%). Conclusively, if first tested by IHC, the 2013 criteria caused more equivocal HER2 IHC cases to be referred to *Her2* FISH testing than the 2007 criteria. That decreased the false-negative rate of HER2 status and increased the detection rates of HER2 positivity in mucinous EOC.

## INTRODUCTION

After carefully excluding metastatic mucinous carcinoma and borderline tumors, primary mucinous epithelial ovarian cancer (EOC) makes up approximately 2% to 4% of all ovarian epithelial carcinomas.^1–3^ To date, the pathogenesis and molecular pathway involved in progression of mucinous EOC are yet unrecognized. The possible mechanisms of carcinogenesis include activation/amplification of oncogene, inactivation of tumor suppressor genes, inhibition of apoptosis, angiogenesis, and so on. Oncogene amplification causes oncoprotein overexpression and promotes tumor growth. HER2 positivity, in which the HER2 receptor is either overexpressed in the protein stage and/or amplified at the genomic level, has accounted for approximately 20% to 30% of breast cancers and 18% to 35% of mucinous EOCs.^4–9^

The success experiences of trastuzumab therapy in breast cancer, gastric, or gastroesophageal junction (GEJ) cancer patients and newly developed anti-HER2 drugs encouraged the investigation of anti-HER2 therapy application in other cancers, including mucinous EOC.^10,11^ However, there is, so far, no consensus in defining the HER2 positivity in mucinous EOC.^12^

Immunohistochemistry (IHC) and fluorescence in situ hybridization (FISH) are still widely used in assessing the HER2 status of clinical specimens. The American Society of Clinical Oncology (ASCO) and College of American Pathologists (CAP) proposed original guidelines to test the HER2 status in breast cancers in 2007 and amended those guidelines in 2013 after concerns were raised about false-positive and false-negative HER2 assessments.^13,14^ Even though pathology communities adopted the 2007 ASCO/CAP algorithms previously, they will soon be familiar with the new 2013 modified rules all over the world. In this study, we aimed to compare both HER2 assay performance characteristics in mucinous EOC using the 2007 and 2013 ASCO/CAP scoring criteria, respectively.

## METHODS

The study materials consisted of 49 cases of mucinous EOC; the characteristics of the TMA derivation were described in our previous report.^9^ All the experimental samples used in this study were de-linked from direct patient identifiers, and the research was conducted according to International Conference on Harmonization guidelines and complied with all applicable regulations for protection of human subjects of research, including review and approval by the Institutional Review Board, Chung-Shan Medical University Hospital, Taichung, Taiwan.

### IHC

The HER2 immunostains were performed on the fully automated Ventana Benchmark XT autostainer using pathway antiHER2/neu rabbit monoclonal antibody (clone 4B5, Ventana Medical Systems, Inc, Arizona USA). HER2 IHC score 3+ breast cancer was used as a positive control. Negative controls were obtained by excluding the primary antibody. The slides were mounted with Permount for microscopic examination, and the images were captured by the NIKON ECLIPSE 50i microscope and NIKON DS-Fi1 Digital Camera System for study comparison.

### FISH

The FISH test was performed by the ABBOTT/Vysis PathVysion *Her2* DNA Probe Kit protocol (Path-Vysion CE Product Description, 4/29/2008). The dual-color FISH consisting of 2 labeled DNA probes was performed on sections cut from the same tissue microarray (TMA) blocks. The LSI HER2 probe that spans the entire *Her2* gene was labeled in Spectrum Orange, and the CEP17 (chromosome-17 centromere; for chromosome-17 enumeration) probe was labeled in SpectrumGreen and hybridized to the alpha satellite DNA located at the centromere of chromosome-17 (17p11.1–q11.1). Counting 2 separate fields of at least 20 cells was essential. We calculated the *Her2*: CEP17 signal ratio by recording the numbers of *Her2* gene (red) and chromosome 17 (green) signals from preselected tumor areas. In most cases, tumor cells from matching sites of IHC analysis were scored for the number of red (*Her2*) and green (CEP17) signals. Signal photos were taken with the NIKON ECLIPSE 80i fluorescent microscope with a PlanFluor oil objective (100×) using a double band-pass filter that permitted simultaneous green and red colors.

### IHC and FISH Interpretation

We applied the 2007 and 2013 ASCO/CAP guideline algorithm for breast tumors to interpret both the results of HER2 IHC and *Her2* FISH tests in this study (Table 1).

**TABLE 1:**
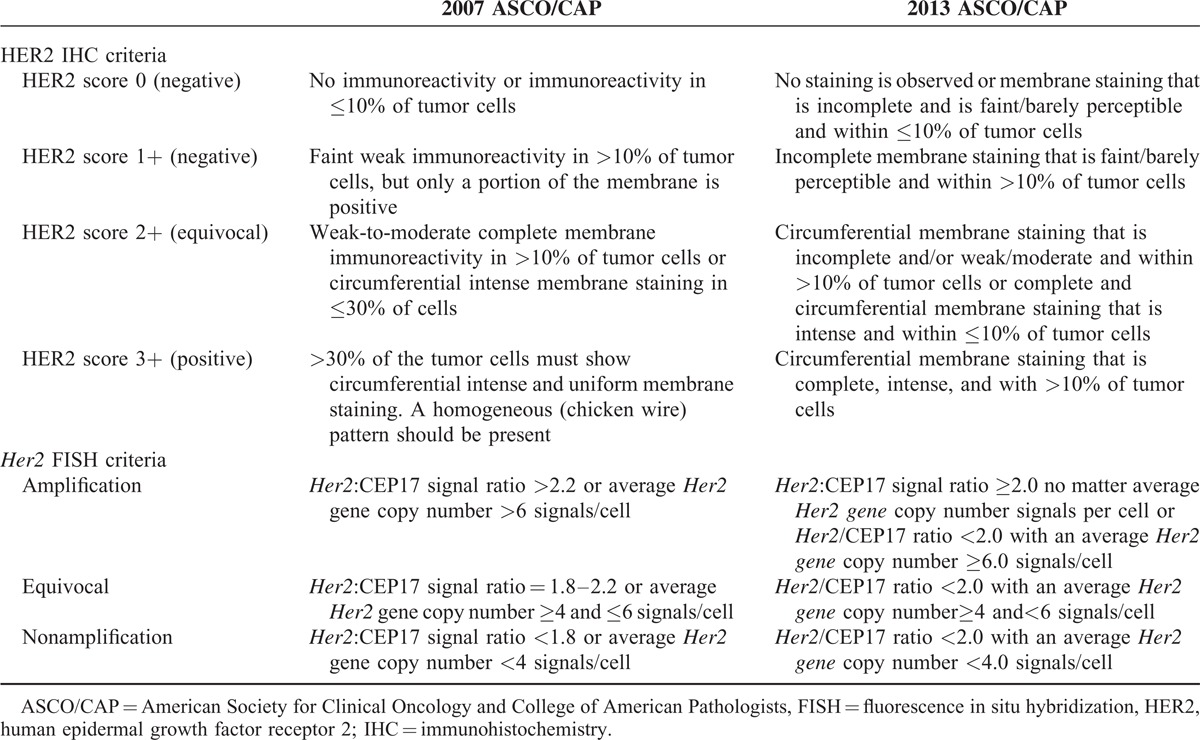
HER2 IHC and *Her2* FISH Criteria Under the 2007 and the 2013 ASCO/CAP Scoring Methods

Our laboratory performance met the proficiency testing requirements of the Taiwan Division of International Academy of Pathology. For our laboratory quality assurance and quality control (QA/QC) assessments, we ran an HER2 control daily in all cases and had 1 pathologist routinely screening the slides.

### Statistical Analysis

The consistency between 2007 and 2013 ASCO/CAP IHC results was analyzed by categorized variables using Kappa statistics. The HER2 positivity was defined as having a positive IHC result irrespective of the FISH ratio, plus equivocal or negative IHC result but FISH amplification. Furthermore, we applied the Cochran-Armitage trend test to assess for a trend of positive percentages across the ordinal variables. Regarding *Her2* FISH as the reference standard, the HER2 IHC performance measures were calculated by 2007 and 2013 ASCO/CAP scoring criteria, respectively. Sensitivity was defined as the ratio of HER2 IHC-positive cases among *Her2* FISH-amplified patients, specificity was defined as the ratio of HER2 IHC-negative cases among *Her2* FISH nonamplified patients, positive predictive value (PPV) was defined as the ratio of *Her2* FISH-amplified cases among HER2 IHC-positive patients, negative predictive value (NPV) was defined as the ratio of *Her2* FISH nonamplified cases among HER2 IHC-negative patients, as well as accuracy was defined as the ratio of HER2 IHC-positive and *Her2* FISH–amplified cases plus HER2 IHC-negative cases and *Her2* FISH nonamplified cases among all cases. The overall concordance was defined as the ratio of HER2 IHC-positive and *Her2* FISH-amplified cases plus HER2 IHC-negative cases and *Her2* FISH nonamplified cases among all nonequivocal IHC cases. Data were analyzed using standard statistical software (SPSS Inc, Chicago, IL). All tests were 2-sided and the significance level was 0.05.

## RESULTS

**FIGURE 1:**
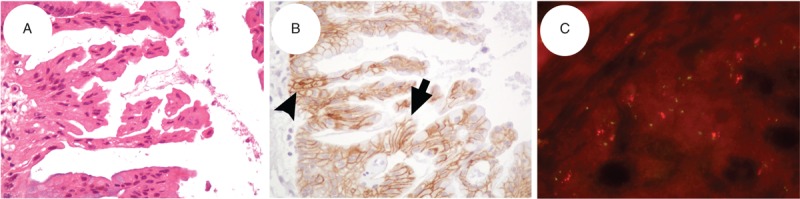
(A) Hematoxylin and eosin stains of a representative case shows that mucinous carcinoma of the ovary consisted of complex glandular proliferation of tumor cells with intracytoplasmic mucin (400×). (B) Immunohistochemical stain: an arrow points to HER2 score 2+ by the 2013 ASCO/CAP criteria (moderate membrane staining that is incompletely circumferential in >10% tumor cells) and score 1+ by the 2007 ASCO/CAP criteria (moderate partial membrane staining that is lateral and basolateral staining >10% tumor cells). Another black arrowhead points to crossly and tangentially cutting glands with the complete and circumferential staining pattern (400×). (B) FISH study shows *Her2* gene amplification with clusters of multiple gene copies in a few tumor cells. *Her2*:CEP17 ratio = 2.89 (5.5/1.9) (1000×). ASCO/CAP = American Society for Clinical Oncology and College of American Pathologists, FISH = fluorescence in situ hybridization.

In this study, a total of 49 specimens of mucinous EOC from Asian women were available for the evaluation of HER2 status. Three cases with weak-to-moderate incomplete membrane staining were classified as having HER2 IHC score 1+ (negative) by the 2007 criteria but reclassified as having HER2 IHC score 2+ (equivocal) by the 2013 criteria (Table 2), while one case of them (n=1/3) had a Her2:CEP17 ratio (5.5/1.9=2.89) that satisfied both the 2007 and the 2013 criteria for Her2 FISH amplification (Figure 1). The other 46 remaining cases showed their HER2 IHC scores unchanged by either the 2007 or the 2013 criteria (Table 2). When comparing the categorical HER2 IHC results (scores 0, 1+, 2+, 3+) by the 2007 and the 2013 ASCO/CAP scoring criteria, they agreed almost perfectly (kappa = 0.903, 95% CI 0.801–1.000) (Table 2). Additionally, when comparing the categorical *Her2* FISH results (non-amplification, equivocal, amplification) by the 2007 and the 2013 ASCO/CAP scoring criteria, they agreed perfectly (kappa = 1.000, 95% CI 1.000∼1.000) (Table 2).

**TABLE 2:**
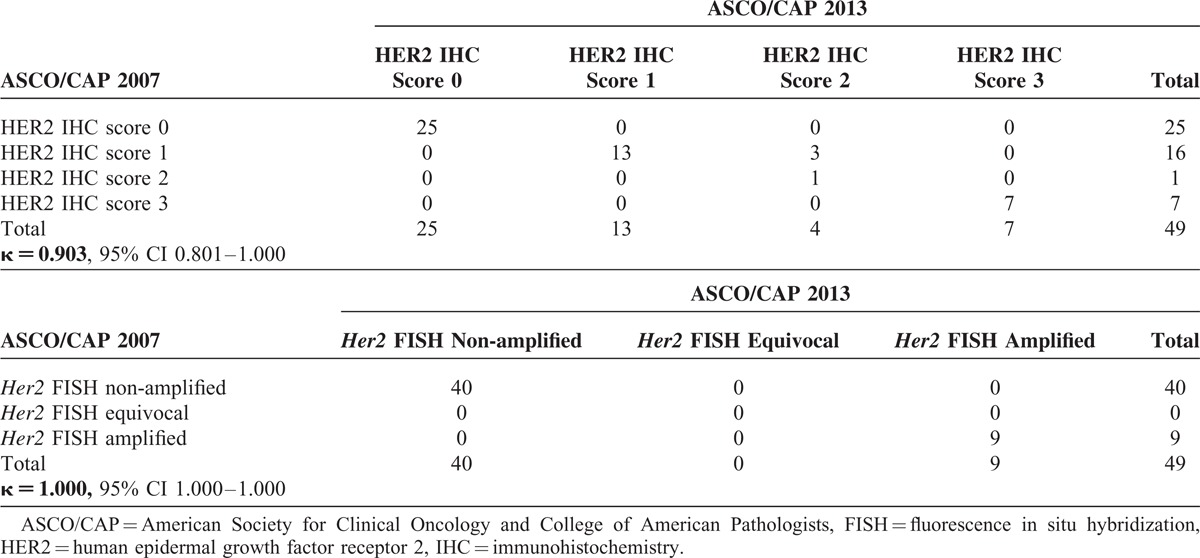
Concordances Between Both HER2 IHC Results and Between Both *Her2* FISH Results Derived From 2007 Versus 2013 ASCO/CAP Criteria by Kappa Statistics

Under the 2007 and the 2013 ASCO/CAP scoring criteria, we demonstrated that *Her2* amplification rates of mucinous EOC (n = 9/49) were the same in both the criteria (18.37% vs 18.37%); the percentage of *Her2* FISH amplification increased significantly in a trend through the ordinals of HER2 IHC results (scores 0, 1+, 2+, 3+) by either the 2007 or the 2013 criteria, respectively (*P* < 0.001 vs *P* < 0.001) (Table 3). We also found that none exhibited *Her2* FISH equivocal results by both the 2007 and the 2013 criteria. Of the four HER2 IHC score 2+ (equivocal) cases which were identified based on the 2013 criteria, two cases show FISH amplification and two cases show FISH nonamplification. On the other hand, only one HER2 IHC score 2+(equivocal) case is identified based on the 2007 criteria and it shows FISH amplification.

**TABLE 3:**
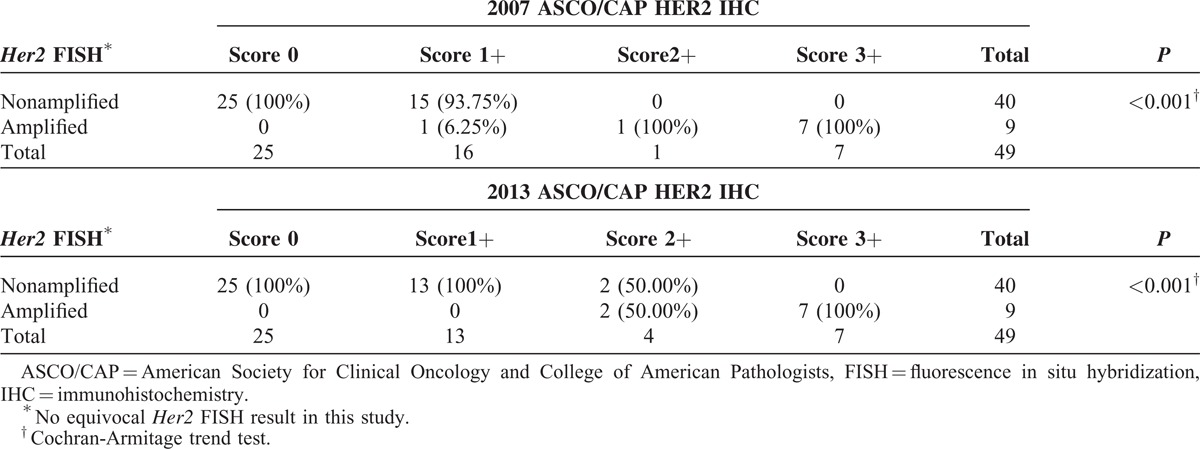
HER2 Status Determined by 2007 and 2013 ASCO/CAP Criteria, Respectively

Except for 1 case with a HER2 IHC score 2+ (equivocal) by the 2007 criteria, our data for the relationship between IHC and FISH showed 100% (n = 7/7) in positive concordance, 97.56% (n = 40/41) in negative concordance, and 97.92% (n = 47/48) in overall concordance (kappa = 0.921, 95% CI 0.769–1.000); whereas except for the 4 cases with HER2 IHC score 2+ (equivocal) by the 2013 criteria, our data for the relationship between IHC and FISH showed 100% (n = 7/7) in positive concordance, 100% (38/38) in negative concordance, and 100% (45/45) in overall concordance (kappa = 1.0000, 95% CI 1.000–1.000) (Table 4).

**TABLE 4:**
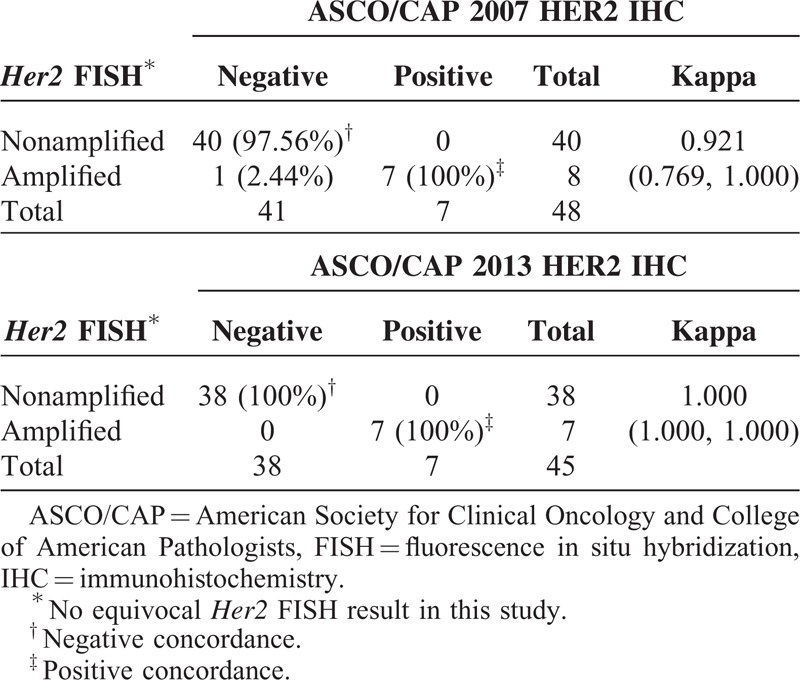
The Agreement Between the Nonequivocal HER2 IHC and *Her2* FISH Under 2007 and 2013 ASCO/CAP Scoring Criteria, Respectively

Using *Her2* FISH as the reference standard, the HER2 IHC performance characteristics under both the 2007 and the 2013 criteria were evaluated by calculation of sensitivity, specificity, PPVs, NPV, and accuracy. After excluding the equivocal cases, the specificity (100%) and PPV (100%) by both the 2007 criteria and the 2013 criteria were similar. The sensitivity under the 2007 criteria was lower than that under the 2013 criteria (87.5% vs 100%), the NPV under the 2007 criteria was lower than that under the 2013 criteria (97.6% vs 100%), and the accuracy under the 2007 criteria was lower than that under the 2013 criteria (97.9% vs 100%) (Table 5).

**TABLE 5:**
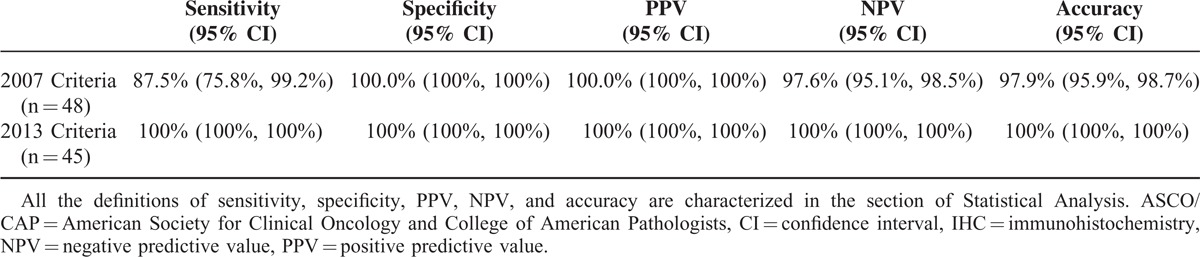
Assay Performance Characteristics of Nonequivocal HER2 IHC Measured by the 2007 and the 2013 ASCO/CAP Criteria, Respectively

## DISCUSSION

We have recently reported the assessment of HER2 status in mucinous EOC on the basis of the 2013 ASCO/CAP guideline update.^9^ However, all thresholds (cutoffs) of positive and equivocal results by HER2 IHC and *Her2* FISH tests have been down-adjusted, and the 2013 criteria seemed to be less stringent than the 2007 criteria (Table 1). In this study, we wished to compare the assay performance characteristics of HER2 status in mucinous EOC by the 2007 and the 2013 ASCO/CAP criteria.^13,14^

The major changes of HER2 scoring methods between the 2013 and the 2007 guidelines are discussed below:

1. The *Her2* gene-amplified cases by FISH include those with *Her2*/CEP17 ratios ≥2.0 in the 2013 criteria versus >2.2 in the 2007 criteria independent of the absolute *Her2* gene copy number, and also include cases with absolute *Her2* gene copy numbers ≥6.0. Our data revealed that 9 cases (n = 9/49) had *Her2* gene amplified by both the 2007 and the 2013 *Her2*/CEP17 thresholds, respectively (>2.2 vs ≥2.0), but none (n = 0/49) had *Her2*/CEP17 <2.2/<2.0 with *Her2* gene copy numbers ≥6.0 by either the 2007 or the 2013 criteria.
2. The equivocal *Her2* FISH test in the 2013 criteria was defined as cases showing a *Her2*/CEP17 <2.0 and an average absolute *Her2* signal count per cell of ≥4.0 and <6.0 versus the 2007 criteria, which defined equivocal as cases showing a *Her2*/CEP17 ratio between 1.8 and 2.2 or *Her2* signal count per cell of ≥4.0 and <6.0. Our data revealed that no equivocal *Her2* FISH cases (n = 0/49) occurred by both the 2007 and the 2013 criteria.
3. The positive HER2 IHC test (score 3+) was defined as circumferential, complete, uniform, and intense staining of >10% of tumor cells in the 2013 criteria versus >30% in the 2007 criteria. Our data revealed that 7 cases (n = 7/49) with HER2 IHC score 3+ existed by both the 2007 and the 2013 criteria. All of them (n = 7) showed homogenous strong, complete membrane staining >30%.
4. The equivocal HER2 IHC result (score 2+) was defined as circumferential membrane staining that is “incomplete” and/or weak/moderate and >10% of tumor cells in the 2013 criteria versus “complete” membrane staining in the 2007 criteria, or complete and circumferential membrane staining that is intense and within ≤10% of tumor cells in the 2013 criteria versus ≤30% in the 2007 criteria. Our data revealed that the 2013 criteria identified more HER2 IHC equivocal cases (n = 4/49, 8.16%) than the 2007 criteria (n = 1/49, 2.04%) in all 49 cases (Table 3).
5. The definition of negative HER2 IHC test (score 0, 1+) was unchanged under 2007 and 2013 criteria. The alterations from HER2 IHC score 0 to 1+ have no clinical relevance. Both the HER2 IHC score 0 and score 1+ have been regarded as “negative” result category. Our data revealed that 25 cases remained HER2 IHC score 0 (negative) and none were upgraded to a score of 1+ by either the 2007 or the 2013 criteria. However, 3 in 16 cases with an IHC score of 1+ (negative) by the 2007 criteria would be reclassified as IHC equivocal (score 2+) result by the 2013 criteria, which therefore required alternative FISH testing (Table 2).

We identified that 2.04% (n = 1/49, if first tested by IHC) or none (n = 0/49, if first tested by FISH) of HER2-positive patients would be missed if the 2007 criteria were used. In other words, if the HER2 IHC test was applied first, the 2013 criteria can detect 1 case with *Her2* FISH amplification in IHC equivocal category, which was classified as IHC-negative category by the 2007 scoring criteria. Compared with the 2013 criteria, the more stringent 2007 criteria inevitably caused false-negative HER2 IHC results, which may take away the opportunity of certain cases to be referred to FISH testing (Figure 1). It means that such cases with *Her2* FISH amplification would be lost by the 2007 criteria, but would not be ignored by the 2013 criteria because the HER2 IHC-negative (score 0, 1+) cases would be considered to have negative HER2 status without any further testing on the basis of the 2007 and 2013 ASCO/CAP algorithms. As a result, the rigorous 2007 ASCO/CAP criteria potentially diminished the detection rates of HER2 positivity in mucinous EOC patients in comparison with the undemanding 2013 ASCO/CAP criteria.

However, the lenient 2013 guidelines may permit more patients to go through the second round of testing, so as to avoid the possibilities of missing HER2 positive mucinous patients. Thus, it unfavorably increases the cost of examination and extends the reporting date of HER2 status.

Even though it was difficult to know in some cases for sure whether the result was positive or negative, leading to so-called “equivocal” results by either IHC or FISH tests, we still decided to choose FISH as the reference standard for *Her2* testing in this study on the mucinous EOC. The major reasons were: FISH has been shown to be theoretically easier to interpret due to the stability of the DNA target; the interobserver variation was lower because FISH was an objectively quantitative test; and none with *Her2* equivocal FISH existed under both 2007 and 2013 criteria in all 49 cases. So that, the assay performance characteristics of HER2 IHC testing by both 2007 and 2013 criteria were assessed and compared (Table 5). Our data favor sensitivity (100%) and NPV (100%) by the 2013 criteria over those (sensitivity 87.5%, NPV 97.6%) by the 2007 criteria. After excluding HER2 IHC equivocal cases, the overall accuracy by 2013 criteria was superior to 2007 criteria (100% vs 97.9%).

The limitations of the TMA study seem to insufficiently reflect the real distribution of a biomarker. Additionally, the issue of intratumoral heterogeneity has been demonstrated and discussed previously.^9,15,16^ We suggest that the percentages of HER2 IHC-positive and *Her2* FISH amplification might be underestimated in the TMA study. However, sampling with optimal cores appeared to be enough to show accuracies compared with whole mount sections.^17,18^

In summary, we demonstrated that both 2007 and 2013 ASCO/CAP scoring criteria agreed excellently in assessing the HER2 IHC (kappa = 0.903) and *Her2* FISH (kappa = 1.000) in mucinous EOC, respectively; both the frequencies of *Her2* FISH amplifications by 2007 and 2013 ASCO/CAP scoring criteria were equivalent (18.37%; n = 9/49); the frequency of HER2 IHC equivocal results by 2007 ASCO/CAP scoring criteria (2.04%; n = 1/49) was less than that by 2013 ASCO/CAP scoring criteria (8.16%; n = 4/49); and 1 case with *Her2* FISH amplification was missing, when first tested by IHC under the 2007 criteria.

## CONCLUSION

When evaluating the HER2 status by IHC first in mucinous EOC, we identified 1 more case with HER2 positivity by the 2013 than that by the 2007 ASCO/CAP guideline scoring criteria. Compared with the 2013 criteria, the more rigorous 2007 criteria resulted in inevitable false-negative IHC, which not only subtracted the opportunity to be referred to FISH testing, but also possibly diminished the detection rates of HER2 positivity in mucinous EOC.
